# Motoric Cognitive Risk Syndrome: Could It Be Defined Through Increased Five-Times-Sit-to-Stand Test Time, Rather Than Slow Walking Speed?

**DOI:** 10.3389/fnagi.2018.00434

**Published:** 2019-01-23

**Authors:** Harmehr Sekhon, Cyrille P. Launay, Julia Chabot, Gilles Allali, Olivier Beauchet

**Affiliations:** ^1^Division of Geriatric Medicine, Department of Medicine, Sir Mortimer B. Davis – Jewish General Hospital and Lady Davis Institute for Medical Research, McGill University, Montreal, QC, Canada; ^2^Division of Experimental Medicine, Faculty of Medicine, McGill University, Montreal, QC, Canada; ^3^Geriatric Medicine and Geriatric Rehabilitation Service, Department of Medicine, Lausanne University Hospital, Lausanne, Switzerland; ^4^Division of Geriatric Medicine, Department of Medicine, St. Mary’s Hospital Center, McGill University, Montreal, QC, Canada; ^5^Department of Neurology, Geneva University Hospital, University of Geneva, Geneva, Switzerland; ^6^Dr. Joseph Kaufmann Chair in Geriatric Medicine, Faculty of Medicine, McGill University, Montreal, QC, Canada; ^7^Lee Kong Chian School of Medicine, Nanyang Technological University, Singapore, Singapore

**Keywords:** older inpatients, epidemiology, screening, cognition, motricity

## Abstract

**Background:** Slow walking speed, time to perform the five-times-sit-to-stand (FTSS) test and motoric cognitive risk syndrome (MCR; defined as slow gait speed combined with subjective cognitive complaint) have been separately used to screen older individuals at risk of cognitive decline. This study seeks to (1) compare the characteristics of older individuals with MCR, as defined through slow walking speed and/or increased FTSS time; and (2) examine the relationship between MCR and its motor components as well as amnestic (a-MCI) and non-amnestic (na-MCI) Mild Cognitive Impairment.

**Methods:** A total of 633, individuals free of dementia, were selected from the cross-sectional “Gait and Alzheimer Interactions Tracking” study. Slow gait speed and increased FTSS time were used as criteria for the definition of MCR. Participants were separated into five groups, according to MCR status: MCR as defined by (1) slow gait speed exclusively (MCRs); (2) increased FTSS time exclusively (MCRf); (3) slow gait speed and increased FTSS time (MCRsaf); (4) MCR; irrespective of the mobility test used (MCRsof); and (5) the absence of MCR. Cognitive status (i.e., a-MCI, na-MCI, cognitively healthy) was also determined.

**Results:** The prevalence of MCRs was higher, when compared to the prevalence of MCRf (12.0% versus 6.2% with *P* ≤ 0.001). There existed infrequent overlap (2.4%) between individuals exhibiting MCRs and MCRf, and frequent overlap between individuals exhibiting MCRs and na-MCI (up to 50%). a-MCI and na-MCI were negatively [odd ratios (OR) ≤ 0.17 with *P* ≤ 0.019] and positively (OR ≥ 2.41 with *P* ≤ 0.019) related to MCRs, respectively.

**Conclusion:** Individuals with MCRf are distinct from those with MCRs. MCRf status does not relate to MCI status in the same way that MCRs does. MCRs is related negatively to a-MCI and positively to na-MCI. These results suggest that FTTS cannot be used to define MCR when the goal is to predict the risk of cognitive decline, such as future dementia.

## Introduction

Motoric cognitive risk syndrome (MCR) is defined as the relationship between objective slow gait speed and subjective cognitive complaint (Verghese et al., 2013). MCR is one of the stages of pre-dementia, similar to mild cognitive impairment (MCI) (Verghese et al., 2013, 2014). MCR does not require a time-consuming comprehensive neuropsychological assessment when compared to MCI, which opens new perspectives in terms of detection of individuals who are at risk of dementia in older populations (Verghese et al., 2013, 2014; Belleville et al., 2017). The past decade has been characterized by an increased interest in identifying and validating biomarkers for early diagnosis and identification of individuals who are at risk of dementia (Belleville et al., 2017). However, the use of biomarkers has limitations in many settings. For instance, access to neuroimaging is difficult and the cost of biological biomarkers limits their use (Handels et al., 2017). Additionally, the highest prevalence and incidence of dementia in the coming years will be observed in low and intermediate income countries, where the accessibility of actual biomarkers is limited (de Jager et al., 2017). Hence, there is a need to optimize and increase the accessibility to clinical risk assessment of dementia in community-dwelling older populations. Using a motor test to predict dementia in older populations may be a solution.

Motoric cognitive risk syndrome has the potential to rapidly screen individuals who are at risk of dementia in a primary care setting, where the under-diagnosis of dementia is estimated to be around 50% in individuals over 65 (Iliffe et al., 2009). This under-diagnosis of dementia is largely related to limited resources and the time required for in-depth assessments of cognitive complaint (Iliffe et al., 2009; Villars et al., 2010). The simplicity of assessment of MCR syndrome could help overcome this issue. However, gait speed, a component of MCR, may be difficult to assess during a primary care visit because of space constraints (Abellan van Kan et al., 2009). Gait speed must be recorded at usual steady state pace rhythm over at least 3 meters (Middleton et al., 2015). Few consultation rooms in primary care possess the features required for the assessment of gait speed, which complexifies the process of consultation and increases physician workload and consult time, when gait speed must be measured. It has been reported that consult time in general practice is very short (around 6.9 min) and depends on the physician, the physician’s workload and the type of visit (Petek Ster et al., 2008). There is, therefore, a need in primary care for a simpler mobility test, which can be completed rapidly and within limited space, so as to facilitate MCR diagnosis in primary settings. In addition, the chosen motor test must be proven to show a link to cognitive impairment or risk of dementia, as the objective of a redefined MCR is to identify individuals at risk of dementia.

The five-times-sit-to-stand test (FTSS) is a physical test, which measures the time taken by an individual to repeat five consecutive chair rises as quickly as possible (Whitney et al., 2005). This motor test examines the challenged balance condition, which is the transfer from a sitting position to a stand-up position. The FTSS test possesses the necessary features for assessment of mobility performance to diagnose MCR in primary care, as it can easily and rapidly be performed in limited space and its requirements are limited to a chair and a stopwatch. In addition, this test may be performed at the time of consultation, as its duration is of fewer less than 2 min in length, including explanation and performance (Whitney et al., 2005). Thus, the FTSS test does not increase the physician’s workload. The one-leg-balance (OLB) test is another simple motor test to examine the challenged balance condition. In it, the individual is asked to stand unassisted on one leg. An impaired OLB test result – defined as being unable to stand on one leg for 5 s – has been identified as a predictor of injurious falls among community-dwelling older adults and cognitive decline in patients with dementia, but not in non-demented individuals (Vellas et al., 1997). In contrast, increased FTSS time has been associated with low cognitive performance in older community dwellers free of dementia (Annweiler et al., 2011). Because non-demented individuals with poor cognitive performance like MCI are at risk of dementia, this association suggests that poor FTSS performance (i.e., increased time) may be used to identify individuals at risk of dementia and thus, that it could be used as an alternate motor test, as opposed to gait speed, to define MCR. Using FTSS performance instead of gait speed to define MCR value for the prediction of dementia requires an investigation, which will determine whether or not individuals classified as MCR through FTSS performance and gait speed are one and the same. This line of questioning is justified, as the FTSS test explores different subdomains of mobility, when compared to gait speed (Whitney et al., 2005; Annweiler et al., 2011; Beauchet et al., 2017; Sekhon et al., 2017). The FTSS test examines the ability to transfer from a sitting position and depends largely on balance control, muscle mass, strength, and the power of lower limbs (Whitney et al., 2005). In comparison, gait speed is a surrogate measure of gait ability, which depends on different body movements and higher levels of movement control involving executive and memory functions (Abellan van Kan et al., 2009; Middleton et al., 2015; Beauchet et al., 2017). These differences between the FTSS test and gait speed, therefore, call into question the possible overlap between individuals whose MCR status was determined using either the FTSS test or gait speed, and their relative MCI.

Motoric cognitive risk syndrome and cognitive impairment are both intermediate stages between normal cognitive aging and major neurocognitive disorders (Verghese et al., 2013, 2014; Belleville et al., 2017). A knowledge gap exists regarding the relationship between MCR and MCI syndromes. Recently, we underscored that there exists overlap between MCR – defined through slow gait speed – and MCI in the population of older community dwellers (Sekhon et al., 2017). The prevalence of MCI was higher in individuals with MCR, when compared to those without MCR (47.2% versus 39.5%) (Sekhon et al., 2017). Unfortunately, the relationship between MCR subcategories of MCI syndromes such as amnestic (a-MCI) and non-amnestic (na-MCI) has not been examined in this study. As gait is largely controlled by executive functions (Beauchet et al., 2017), we have hypothesized that MCR as defined by slow gait speed (MCRs) may be more frequently associated with na-MCI, when compared to MCR as defined by increased FTSS time (MCRf), which may be associated with a-MCI. Using the data of the cross-sectional study known as the “Gait and Alzheimer interactions tracking” (GAIT) study (Beauchet et al., 2018), we had the opportunity to explore the overlap between MCR as defined by slow gait speed and increased FTSS time, and their relationship with a-MCI and na-MCI. This study aims to (1) compare the characteristics of participants of the GAIT study with and without MCR as defined by slow gait speed and increased time of FTSS, and (2) examine the relationship between MCR, and a-MCI and na-MCI. Comparing gait speed and FTSS as a construct of MCR, as well as their relationship with MCI subtypes, may provide new insight into the interaction between motor and cognitive impairment in the aging population.

## Materials and Methods

### Population and Study Design

A subgroup of older individuals recruited in the GAIT study were selected for the present study. The GAIT study is a cross-sectional design-based study, which was conducted in France between November 2009 and 2015 (Beauchet et al., 2018). All GAIT participants were relatively healthy community-dwelling individuals, who were recruited during a visit in the memory clinic of Angers University Hospital, in France, for cognitive complaint evaluation. The GAIT study inclusion criteria were: (1) age 65 years and over, (2) living at home in the community, and (3) an adequate understanding of French. Exclusion criteria included acute medical illness (regardless of nature) in the past month; extrapyramidal rigidity of the upper limbs (regardless of etiology); neurological diseases [past history of stroke, (NPH), multiple sclerosis, Parkinson’s disease, cerebellar disease, polyneuropathy, and vestibular disease]; psychiatric diseases (past history of psychosis, personality disorders or severe depression as well as active depression as defined by a 4-item geriatric depression scale (GDS) score above 1) (Shah et al., 1997) other than cognitive impairment; severe gait-affecting medical conditions which left potential participants with the inability to walk unassisted for 15 min, such as rheumatologic diseases (spine, pelvic, and joint arthritis with deformation); and ophthalmic diseases with severe vision abnormality. In addition, for the present study, we also excluded participants with NPH or presenting vascular brain abnormalities (i.e., lacunar lesions and strokes) on brain imaging [i.e., computed tomography (CT) or magnetic resonance imagery (MRI) scan] performed during the assessment, suffering from dementia, using a walking aid, and presenting no gait speed or FTSS time data. A total of 663 participants were selected, after applying these selection criteria.

### Study Assessments

The selected GAIT participants had a full-standardized clinical examination, a comprehensive neuropsychological assessment, brain imaging (i.e., MRI or CT) and blood tests including Vitamin B12, TSH, calcemia and other serum electrolytes, creatinine, and urea. Age, sex, educational level [evaluated by number of years of schooling and categorized by high school level (i.e., yes or no)], number of drugs taken daily and body mass index (BMI; kg/m^2^) were recorded. Maximal isometric voluntary contraction (MVC) strength of hand was measured with the help of a computerized hydraulic dynamometer (Martin Vigorimeter, Medizin Tecnik, Tutlingen, Germany). The test was performed once on each side. The highest MVC value recorded was used in the present data analysis. Binocular distance vision was measured at 5 m with a standard Monoyer letter chart and scored from 0 (i.e., worst performance) to 10 (i.e., best performance) (Lord et al., 1991b). Vision was assessed with corrective lenses if needed. Lower-limb proprioception was evaluated with the help of a graduated tuning fork placed on the tibial tuberosity, so as to measure vibration threshold (Buchman et al., 2009). The mean value obtained for the left and right sides ranged between 0 (i.e., worst performance) and 8 (i.e., best performance) and was used in the present data analysis. Gait speed was measured with the help of GAITRite^®^ (Gold walkway, 972 cm long, active electronic surface area 792 cm × 610 cm, total of 29,952 pressure sensors, scanning frequency 60 Hz, CIR System, Havertown, PA, United States). Time to perform FTSS was also measured. A trained evaluator demonstrated the test procedure while giving standardized verbal instructions. Moreover, before testing, participants were allowed to practice the sit-to-stand test twice. Participants began by crossing their arms upon their chest and sitting with their back against the chair (45 cm above the floor). The chair was padded and armless. They were prompted not to bounce off the chair when returning to the standing position, and reminded to fully straighten their legs when elevating. Participants were instructed to stand up and sit down five times as quickly as possible. Performance was measured with a stopwatch in seconds, from the time at initial seated position to the time at final seated position, after completing five stands. A bedside face-to-face neuropsychological assessment was also performed using the mini-mental state examination (MMSE) (Folstein et al., 1975) and frontal assessment battery (FAB) (Dubois et al., 2000), the French version of the free and cued selective reminding test-total recall (FCSRT-TR) (Van der Linden et al., 2004), parts A and B of the trail making test (TMT) (Brown et al., 1958), the Stroop Test (Stroop, 1935), and the instrumental activities of daily living scale (IADL) (Pérès et al., 2006). The diagnosis was determined, following a standardized procedure and consensual definition, during multidisciplinary meetings involving geriatricians, neurologists, and neuropsychologists of Angers University Memory Clinic. It was based on the aforementioned neuropsychological tests, physical examination findings, blood tests, and MRI or CT scan of the brain. First, the cognitive status was determined using the performances obtained during the neuropsychological assessment. Participants were classified within cognitively healthy individuals (CHI), a-MCI and na-MCI categories, diagnosis of MCI being in accordance with the criteria detailed by Dubois et al. (2010). CHI were individuals who exhibited normal cognitive function with all cognitive scores using the referent age-appropriate mean value. Participants with a-MCI and na-MCI were individuals, who have an objective impairment in the memory (i.e., a-MCI) or non-memory (i.e., na-MCI) domains, respectively, defined as a score >1.5 SDs beneath the age-appropriate mean, and who have not impaired daily living activities (i.e., normal IADL score). Second, the etiology of MCI (i.e., related to neurodegenerative brain lesions versus secondary to metabolic disorders) was determined using the results of blood tests and the brain MRI.

### Definition of Motoric Cognitive Risk Syndrome and Categorization of Participants

Different definitions of MCR were used for each subgroup. First, the diagnosis of MCR was made through slow walking speed (MCRs) in accordance with the criteria described by Verghese et al. (2013): a combination of cognitive complaint and slow gait, with the absence of dementia or any mobility disability. As cognitive complaint was the reason for referral to the memory clinic for participants of the GAIT study, all of them met the criteria for cognitive complaint. Slow gait speed was defined as gait speed of one SD or greater, beneath the age-and sex-appropriate mean values established by the present cohort, as done in previous studies (Verghese et al., 2013, 2014). Second, MCR was also defined using increased FTSS time (MCRf) defined as time one SD or greater, above the age-and sex-appropriate mean values established by the present cohort. Five subgroups of individuals were identified: (1) those with MCRs using gait speed exclusively; (2) those with MCRf using FTSS time exclusively; (3) those with MCR with abnormal scores in both gait speed and FTSS time (MCRsaf); (4) those with MCR irrespective of mobility test used (MCRsof); and (5) those without MCR.

### Standard Protocol Approvals, Registrations, and Patient Consent

This study was conducted in accordance with the ethical standards set forth in the Helsinki Declaration (1983). Participants in the study were included after obtaining written informed consent for research. The local Angers Ethics Committee approved the study protocol (n°2009-A00533-54).

### Statistics

The participants’ characteristics were summarized using means and SDs or frequencies and percentages, as appropriate. Between-group comparisons were performed using a Kruskal-Wallis or Chi square test, Mann-Whitney, independent *t*-test; unpaired *t*-test or Chi square test, as appropriate. Uni and multiple logistic regression analyses were performed to examine the relationship between MCR (i.e., dependent variable) and MCI (i.e., independent variable), relative to participants’ characteristics. *P*-values less than 0.05 were considered statistically significant. All statistics were performed using SPSS (version 23.0; SPSS, Inc., Chicago, IL, United States).

## Results

Table 1 illustrates the participants’ characteristics and their comparisons between the different subgroups of participants based on MCR definition. A total of 76 (12.0%) participants were classified as having MCRs, 39 (6.2%) MCRf, 15 (2.4%) MCRsaf, and 130 (20.5%) MCRsof. Individuals with MCR, irrespective of the type of MCR, had the same clinical characteristics, except for sex and level of education. Prevalence of women varied between the different subgroups of MCR (*P* = 0.029), the highest prevalence being observed with MCRs. Participants with MCRs displayed a lower level of education when compared to those with MCRf (*P* = 0.008). Participants with MCR displayed lower limb proprioception when compared to non-MCR participants (*P* = 0.042). The prevalence of MCI syndrome, regardless of type, was significantly different between the three subgroups of MCR (*P* = 0.039). The prevalence of a-MCI was lower in individuals with MCRs when compared to those with MCRf (*P* = 0.010) and MCRsaf (*P* = 0.018), whereas the prevalence of na-MCI was higher in individuals with MCRs when compared to those with MCRf (*P* = 0.010) and MCRsaf (*P* = 0.018). Those displaying MCRf registered greater walking speeds when compared to those with MCRs (*P* ≤ 0.001) and MCRsaf (*P* ≤ 0.001). Time to perform FTSS was lower in individuals with MCRs (*P* ≤ 0.001) and MCRsaf (*P* ≤ 0.001). Comparisons between individuals with MCR, irrespective of definition, and without MCR show that all characteristics differed significantly (*P* < 0.05), except for age.

**Table 1:** Comparison of participants’ characteristics according to motoric cognitive risk syndrome definitions (*n* = 633).

	Non-MCR (*n* = 503)	MCR (*n* = 130)					*P*-value			
		Walking speed^∗^ (*n* = 76)	FTSS^∗^ (*n* = 39)	Walking speed and FTTS (*n* = 15)	Walking speed and/or FTTS (*n* = 130)	Overall†	Walking speed versus FTSS‡	Walking speed versus walking speed and FTTS‡	FTSS versus walking speed and FTTS‡	MCR# versus non-MCR¶
Age, mean ± SD (y)	71.8 ± 4.5	71.3 ± 3.3	72.7 ± 4.4	72.8 ± 5.6	71.9 ± 4.0	0.330	0.148	0.464	0.977	0.849
Female, *n* (%)	215 (42.7)	28 (36.8)	9 (23.1)	3 (20.0)	40 (30.8)	**0.029**	0.135	0.208	0.808	**0.013**
Number of drugs daily taken, mean ±*SD*	2.7 ± 2.6	4.5 ± 4.0	3.1 ± 2.8	3.9 ± 3.0	4.0 ± 3.6	0.194	0.072	0.808	0.343	**≤0.001**
Body masse index, mean ±*SD* (kg/m^2^)	25.6 ± 3.5	28.7 ± 5.6	27.2 ± 3.5	28.1 ± 3.7	28.2 ± 4.9	0.413	0.251	0.773	0.255	**≤0.001**
Education level ^∗∗^, *n* (%)	337 (67)	29 (38.2)	25 (64.1)	8 (53.3)	62 (47.7)	**≤0.001**	**0.008**	0.274	0.467	**≤0.001**
Handgrip strength †† (N.m^-2^), mean ±*SD*	32.2 ± 9.3	32.1 ± 8.4	31.9 ± 8.8	32.4 ± 10.1	32.2 ± 8.7	0.771	0.901	0.406	0.416	0.745
Distance vision acuity ‡‡ (/10.9040), mean ±*SD*	8.1 ± 1.6	7.9 ± 1.6	8.0 ± 1.5	7.8 ± 1.3	7.9 ± 1.5	0.268	0.917	0.624	0.641	0.113
Lower-limb proprioception score ¶¶(/8), mean ±*SD*	5.2 ± 1.5	5.0 ± 1.1	4.9 ± 1.7	4.8 ± 1.6	4.9 ± 1.3	**0.048**	0.843	0.904	0.903	**0.042**
MMSE (/30), mean ±*SD*	28.0 ± 1.6	27.2 ± 2.1	27.7 ± 2.1	27.3 ± 1.8	27.4 ± 2.1	0.228	0.093	0.895	0.298	**≤0.001**
FAB (/18), mean ±*SD*	16.3 ± 1.5	15.4 ± 1.8	16.0 ± 1.8	14.6 ± 2.8	15.5 ± 2.0	0.094	0.062	0.422	0.090	**≤0.001**
Mild cognitive impairment, *n* (%)										
All categories	208 (41.4)	40 (52.6)	21 (53.8)	10 (66.7)	71 (54.6)	**0.039**	0.902	0.318	0.393	**0.007**
Amnestic	57 (11.3)	2 (2.6)	6 (15.4)	3 (20.0)	11 (8.5)	**0.023**	**0.010**	**0.018**	0.935	**0.044**
Non-amnestic	151 (30.0)	38 (50.0)	15 (38.5)	7 (46.7)	60 (46.2)	**0.025**	**0.010**	**0.018**	0.935	**0.049**
Walking speed, mean ±*SD* (m/s)	115.3 ± 17.7	82.0 ± 9.5	108.1 ± 16.6	76.2 ± 13.8	89.2 ± 17.7	**≤0.001**	**≤0.001**	0.094	**≤0.001**	**≤0.001**
FTSS, mean ±*SD* (s)	12.6 ± 2.6	13.6 ± 2.4	19.8 ± 2.9	20.9 ± 3.9	16.3 ± 4.2	**≤0.001**	**≤0.001**	**≤0.001**	0.401	**≤0.001**

*MCR, motoric cognitive risk syndrome; FTTS, five times sit-to-stand; SD, standard deviation; MMSE, mini mental status examination; FAB, frontal battery assessment; ^∗^, exclusive; ^∗∗^, high school level (i.e., only participants with mean value of the motor test below 1 standard deviation); †, comparisons between the different subgroups of MCR based on Kruskal-Wallis or Chi square test, as appropriate; ‡, based on Mann-Whitney or Chi square test; #, all subgroups of MCR merged together; ¶, based on independent *t*-test; ‡‡, mean value of the highest value of maximal isometric voluntary contraction strength measured with computerized dynamometers expressed in Newton per square meter; ‡‡, binocular vision acuity at distance of 5 m with a standard Monoyer letter chart; ¶¶, mean value of left and right side and based on graduated diapason tuning fork placed on the tibial tuberosity; *P*-value significant (i.e., *P* < 0.05) indicated in bold*.

Multiple logistic regressions have shown a positive relationship between MCRs and a-MCI and a more marked (Table 2) negative relationship between MCRs and na-MCI (*P* ≤ 0.020). All MCR, irrespective of definition, displayed a positive relationship with MCI (all categories *P* = 0.010, a-MCI *P* = 0.040 and na-MCI *P* = 0.046). These last relationships remained insignificant when logistic regressions were adjusted for all participant characteristics.

**Table 2:** Logistic regressions showing the association between motoric cognitive risk syndrome and mild cognitive impairment (*n* = 633).

			OR [95%CI] *P*-value^∗^					
	
	Walking speed†		FTSS†		Walking speed and FTTS		Walking speed and/or FTTS	
	Model 1	Model 2	Model 1	Model 2	Model 1	Model 2	Model 1	Model 2
**Mild cognitive impairment**					
All categories	1.60	0.99	1.40	1.29	2.34	1.87	–	1.95
	[0.97;2.12]	[0.57;1.71]	[0.69;2.64]	[0.63;2.64]	[0.77;7.11]	[0.59;5.94]	[1.14;2.53]	[0.78;1.89]
	0.064	0.965	0.383	0.485	0.134	0.287	**0.010**	0.385
Amnestic	0.13	0.17	1.23	1.60	1.34	1.72	0.47	0.63
	[0.03;0.57]	[0.04;0.75]	[0.45;3.32]	[0.56;4.56]	[0.34;5.40]	[0.39;7.55]	[0.23;0.97]	[0.30;1.34]
	**0.007**	**0.019**	0.686	0.578	0.677	0.475	**0.040**	0.232
Non-amnestic	2.70	2.40	0.90	0.78	0.86	0.76	1.44	1.25
	[1.31;5.59]	[1.15;5.04]	[0.54;1.47]	[0.46;1.32]	[0.43;1.72]	[0.36;1.60]	[1.01;2.06]	[0.86;1.82]
	**0.007**	**0.020**	0.664	0.358	0.666	0.469	**0.046**	0.249

*OR, odd ratio; CI, confidence interval; MCR, motoric cognitive risk syndrome; FTTS, five times sit-to-stand; ^∗^, separate model for gait speed; FTSS, gait speed and FTTS, gait speed and/or FTTS; †, Exclusive (i.e., only participants with mean value of the motor test below 1 standard deviation); Model 1, adjusted for age and sex; Model 2, adjusted for age, sex, number of drugs daily taken, body mass index, educational level, handgrip strength, distance vision acuity, and lower-limb proprioception. *P*-value significant (i.e., *P* < 0.05) indicated in bold*.

## Discussion

The study findings demonstrate that the use of gait speed and FTSS time to define MCR results in the selection of different subgroups of individuals, with infrequent overlap (2.4%). In contrast, there existed significant overlap between MCR and na-MCI participants (up to 50%). In addition, only MCRs exhibited a significant relationship with the MCI subgroups, the na-MCI subtype, relating positively and the a-MCI relating negatively to this MCR subgroup.

There existed infrequent overlap between individuals with MCR as defined by gait speed and FTSS time. This result suggests that impaired performance in these two motor tests tracks different clinical phenotypes of individuals. But it is not consistent with a previous study, which used alternate gait parameters to define MCR and reported greater overlap (68%) (Allali et al., 2016b). Comparatively, in the previous study the definition used for the different subtypes of MCR involved low performance of gait parameters (mean and variability of stride length and swing time). In our study, even if both gait speed and FTSS examine a condition of dynamic balance in which the body’s center of gravity is maintained within a narrow base of support while moving (Lord et al., 1991a; Dubost et al., 2005), they relate to different brain regions, which may explain the infrequent overlap observed (Lord et al., 1991a; Nutt et al., 1993; Dubost et al., 2005; Rosano et al., 2007; Wittenberg et al., 2017). For instance, gray matter volumes in the left frontal lobe were correlated with usual gait speed in healthy older adults, whereas reduced volumes in putamen and superior posterior parietal lobule were associated with balancing difficulty in semi-tandem stance (Rosano et al., 2007). Functional brain imagery study findings point to involvement of the premotor, supplementary motor, and parietal cortex in standing balance control, whereas the hippocampus and premotor cortex are the key region for gait control (Janssen et al., 2002; Rosano et al., 2007; Beauchet et al., 2009, 2012, 2016; Spyropoulos et al., 2013; Wittenberg et al., 2017). Subcortical regions have also been identified as key regions for gait control including the cerebellar locomotor region, the mesencephalic locomotor region, and the subthalamic locomotor region (Bohnen et al., 2011). Gait speed is a surrogate measure of gait, which is the medical term used to globally describe the human locomotor movement of walking (Nutt et al., 1993; Beauchet et al., 2017). Gait is a complex movement in terms of biomechanics and motor control (Nutt et al., 1993; Rosano et al., 2007; Beauchet et al., 2009, 2012; Wittenberg et al., 2017). It has been highlighted that even the simplest walking condition, such as straight-line walking at a comfortable steady-state pace without any disturbances, involves cortical networks and cognitive functions (Nutt et al., 1993; Rosano et al., 2007; Beauchet et al., 2009; Wittenberg et al., 2017). This association may explain the predictive value of slow gait for the occurrence of dementia (Beauchet et al., 2016). In contrast, FTSS time explores the performance of body transfer movement from a seated position (Whitney et al., 2005). This movement is more unstable in terms of biomechanics, when compared to walking at a comfortable steady-state pace without any disturbances (Spyropoulos et al., 2013). It involves an unstable movement from a static and stable position to a quasi static position (Janssen et al., 2002; Whitney et al., 2005; Bohnen et al., 2011; Schofield et al., 2013; Spyropoulos et al., 2013; Lee et al., 2017). Thus, FTSS time is strongly related to several physiological sensory and motor subsystems which contribute to the dynamic postural control, the most important ones identified in older adults being the muscle strength, lower-limb proprioception, vestibular, and vision subsystems (Janssen et al., 2002; Bohnen et al., 2011; Schofield et al., 2013; Spyropoulos et al., 2013). Balance control like gait control deteriorates with the progression of dementia (Lee et al., 2017). This is similar to the decline of gait control with the progression of dementia (Annweiler et al., 2011). Increased FTSS time has been associated with low cognitive performance in older adults free of dementia (Annweiler et al., 2011). This association has mainly been reported through bedside cognitive tests exploring global cognitive functioning, such as the MMSE the modified mini mental state (3MS) and Pfeiffer’s Short Portable Mental State Questionnaire (Hirsch et al., 1997; Raji et al., 2002; Rosano et al., 2005; Annweiler et al., 2011).

The second main finding of our study is the significant overlap between MCI and MCR, irrespective of the criteria for MCR definition. The prevalence of MCI was significantly higher in individuals with MCR when compared to those without MCR, and ranged from 52.6% for individuals with MCRs to 66.7% for individuals with MCRsaf. In addition, the overlap between MCR and MCI was greater for na-MCI when compared to aMCI. This result concords with our previous study (Sekhon et al., 2017) and underscores the strong relationship between MCR and impaired cognitive performance, which explains the ability for both syndromes to predict dementia (Verghese et al., 2013, 2014; Beauchet et al., 2016; Sekhon et al., 2017). Cognition and locomotion are two human abilities, which are controlled by the brain (Nutt et al., 1993; Beauchet et al., 2009, 2012, 2016). Their decline is highly prevalent with physiological and pathological aging, and is greater than the simple sum of their respective prevalence, suggesting complex age-related interplay between cognition and locomotion (Nutt et al., 1993; Rosano et al., 2007; Beauchet et al., 2009, 2012, 2016; Wittenberg et al., 2017). Recently, a systematic review and meta-analysis provided evidence that poor gait performance could predict dementia (Beauchet et al., 2016). We have previously reported that individuals who exhibited both syndromes had poorer cognitive performance in all domains when compared to participants with MCI without MCR, and to participants with isolated MCR (Sekhon et al., 2017).

Furthermore, the present study concludes that MCR related positively to na-MCI and negatively to a-MCI. This result may be related to studies that reported executive dysfunction in individuals with MCR (Kumai et al., 2016; Belleville et al., 2017; Sekhon et al., 2017). This correlation between MCRs and na-MCI (but not with a-MCI) suggests that in our cohort (i.e., memory clinic based), MCRs is associated with an underlying non-AD process, such as vascular dementia or dementia with Lewy bodies, but not with an underlying AD process. This double dissociation is supported by the observation that at disease onset, gait speed is more affected in non-AD dementia than in AD dementia (Allali et al., 2016a). The absence of relationship between FTSS time and MCI status suggests that there is no interaction with cognitive performance in cognitively impaired individuals, such as MCI individuals. This result was not expected because of the previous positive relationship reported in CHI (Hirsch et al., 1997; Raji et al., 2002; Rosano et al., 2005; Annweiler et al., 2011), which underlines a non-linear complex relation between FTSS time and decline in cognitive performance. This relationship between MCRs and na-MCI supports that MCR appears to be a good predictor of non-Alzheimer’s dementia, and in particular of vascular dementia (Verghese et al., 2013, 2014). Furthermore, the absence of any relationship with MCRf suggests that increased FTSS tracks a profile for older adults which is not relevant in identifying older adults who are at risk of dementia.

Our study has certain limitations. First, the cross-sectional design does not allow us to make any causal association. Secondly, the recruitment of participants was performed in one center. Thirdly, all participants in this study presented a cognitive complaint, preventing the generalization of study findings to all non-demented community-dwelling older adults. Indeed, the non-MCR/non-MCI participants cannot be considered as strictly cognitively intact, but as participants with subjective cognitive impairment (SCI). SCI is a prodromal state of MCI and is considered the earliest clinical stage of dementia (Jessen et al., 2014). Fourthly, we have no brain imaging data on the subset of GAIT participants selected for this study.

## Conclusion

The findings revealed that individuals with MCRf are distinct from those with MCRs. MCRf status does not relate to MCI status in the same way that MCRs does. A-MCI related negatively to MCRs, whereas it related positively to na-MCI. All these results suggest that using FTSS time in the definition of MCR is not appropriate in order to identify older adults who are at risk of dementia.

## Author Contributions

HS and OB studied the concept and design. OB and CL acquired the data. HS, CL, GA, and OB analyzed and interpretated the data. HS drafted the manuscript. CL, JC, GA, and OB critically revised the manuscript for important intellectual content.

## Conflict of Interest Statement

The authors declare that the research was conducted in the absence of any commercial or financial relationships that could be construed as a potential conflict of interest. The handling Editor declared a past co-authorship with several of the authors GA and OB.
